# Therapeutic effects of *Bombax ceiba* flower aqueous extracts against loperamide-induced constipation in mice

**DOI:** 10.1080/13880209.2022.2157841

**Published:** 2022-12-29

**Authors:** Liuping Wang, Shiyuan Xie, Xuan Jiang, Caini Xu, Youqiong Wang, Jianfang Feng, Bin Yang

**Affiliations:** a College of Pharmacy, Jiangxi University of Traditional Chinese Medicine, Nanchang, China; b College of Pharmacy, Guangxi University of Chinese Medicine, Nanning, China; c Guangxi Superior Chinese Patent Medicine and National Medicine Development Engineering Technology Research Center, Nanning, China; d College of Pharmacy, Guangxi Medical University, Nanning, China

**Keywords:** Motilin, gastrin, substance P, somatostatin, AQP_3_, c-kit

## Abstract

**Context:**

*Bombax ceiba* Linnaeus (Bombacaceae) is known as silk cotton tree, the flowers of which are used in many medicinal applications.

**Objective:**

To investigate the therapeutic effect of *B. ceiba* flower aqueous extracts (BCE) against loperamide-induced constipation and characterize the chemical composition of BCE.

**Materials and methods:**

Sixty male Kunming mice were divided into control (saline), model (10 mg/kg loperamide + saline), phenolphthalein (10 mg/kg loperamide + 10 mg/kg phenolphthalein) and different dosage of BCE (10 mg/kg loperamide + 40, 80 and 160 mg/kg BCE, respectively) groups, and received intragastric administrations for eight days. Faecal water content, number of faeces, first black-stool defecation time and gastrointestinal transit rates were evaluated. Various biochemical and molecular biomarkers were assessed in blood and colon. UPLC-ESI-QTOF-MS/MS was used to tentatively identify the composition of the BCE.

**Results:**

BCE treatment (160 mg/kg) could increase faecal water (15.75%), faeces number (11.65%), gastrointestinal transit rate (25.37%) and decrease first black-stool defecation time (24.04%). The BCE (80 mg/kg) increased the serum level of motilin (30.62%), gastrin (54.46%) and substance P (18.99%), and decreased somatostatin (19.47%). Additionally, the BCE (160 mg/kg) reduced the mucosal damage, restored colonic goblet cell function, down-regulated the protein expression of AQP_3_ (33.60%) and increased c-kit protein expression (11.63%). Twelve known compounds, including protocatechuic acid, chlorogenic acid and rutin, previously reported in *B. ceiba,* were identified in the BCE.

**Discussion and conclusions:**

This study suggested that BCE is a promising agent for the treatment of constipation.

## Introduction

Constipation is a clinically common digestive disease in modern society (Chen et al. 2019; Lu et al. 2021), characterized by difficult excretion, low-frequency defecation, hard-dry stool and prolonged gastrointestinal emptying time (Li et al. 2020). Previous surveys showed that the prevalence of constipation ranged from approximately 2–30%, especially in elderly populations and children (Zhao et al. 2019; Gan et al. 2020). Long-term constipation may cause not only feelings of discomfort (Lu et al. 2021), but also gastrointestinal nerve dysfunction, intestinal flora disturbances (Wang et al. 2017), hepatic encephalopathy, cardiocerebrovascular disease (Payne and Grimm 2017), and even fatal pulmonary embolism (Yin et al. 2018) or colorectal cancer (Gan et al. 2020). The cause of constipation may have various factors, including a low-fibre diet, low physical activity, low water intake, medicine abuse (Hayeeawaema et al. 2020), lifestyle changes (Wang et al. 2017). Constipation is also one of the most common non-motor symptoms of Parkinson’s disease, and the most common gastrointestinal symptoms in alimentary tract tumour, inflammatory bowel disease, diabetes, metabolic and endocrine disorders, hyperparathyroidism, hypothyroidism and other neurological diseases (Gao et al. 2021). At present, the pathogenesis of constipation may include changes in gastrointestinal peptides hormones and aquaporins, colonic dysfunction, and abnormal interstitial cells of Cajal (ICC) (Liu et al. 2020). The gastrointestinal tract can produce a variety of gastrointestinal peptides hormones, including inhibitory transmitters represented by somatostatin (SS) and excitatory transmitters represented by motilin (MTL) (Liu et al. 2020), gastrin (Gas), substance P (SP), etc. (Han 2013; Gan et al. 2020). Aquaporin 3 (AQP_3_) is an important aquaporin located in the colon, which play an important role in the water transport effect (Ikarashi et al. 2016). In addition, some studies have shown that ICC is a pacemaker of the intestine (Han et al. 2010; Liu et al. 2020) and constipation is associated with loss and injury to ICC in the gastrointestinal tract (Huizinga and Chen 2014). The stem cell factor and its receptor tyrosine kinase (SCF/C-kit) signalling pathway could affect the phenotype of ICC and it may provide an important target for the therapy of constipation (Li et al. 2019).

More than 50% of patients fail to be cured, though the high global expenditure of constipation therapy annually (Zhang et al. 2021). Administration of laxatives and prokinetic agents (MTL agonists, 5-hydroxytryptamine modulators, opioid antagonists and chloride-channel activators) are common clinical treatments for constipation (Shin et al. 2014). However, long-term intake of these agents can be associated with complications, such as drug dependence, severe diarrhoea (Lu et al. 2021) hypotension, tachycardia, postural dizziness, melanosis coli, etc. (Ma et al. 2019). Therefore, it is urgently needed to find a new drug of hazard-free treatment for constipation. As an alternative treatment for constipation, traditional Chinese medicine is universally applied, safe and effective (Sun et al. 2020). Moreover, some studies suggest that multiple plant extracts could be applied to control constipation and there is hardly any side effect (Gilani et al. 2000; Han 2015; Lu et al. 2021).

*Bombax ceiba* Linnaeus (Bombacaceae) is known as silk cotton tree (Arafa et al. 2019) and it is mainly cultivated in Southern China, India, Pakistan, Egypt and Northern Australia (Xu et al. 2017). Phytochemical investigations have revealed that this plant contains health-promoting phytopharmaceuticals such as naphthoquinones, flavonoids, sesquiterpenoids, phenolics, neolignans and steroids, possessing the medicinal properties (Xu et al. 2017). Furthermore, this plant has a strong ethnobotanical background. It has been used extensively in both Indian and Chinese traditional herbal medicines for the treatment of fever, inflammatory conditions, catarrhal affection (Yu et al. 2011), diuretic, oedema, hepatotoxicity and ulcer (Shahat et al. 2003; Xu et al. 2017). Moreover, according to the traditional Chinese medicine theory, the flower of *B. ceiba* is cool-natured (Zhang et al. 2015), and is regarded as having laxative properties (Shahat et al. 2003).

However, there have been no reports on the therapeutic effects of *B. ceiba* flower aqueous extracts (BCE) in constipation and its relevant mechanisms. Loperamide has been widely used for introducing spastic constipation model (Mori et al. 2013; Li et al. 2015). Therefore, we investigated the curative effect of BCE on loperamide-induced mice and revealed its mechanism by measuring the serum gastrointestinal peptides hormones concentration and the expression of AQP_3_ and receptor tyrosine kinase (c-kit) in the colon. In addition, an ultra-performance liquid chromatography coupled with electrospray ionization quadrupole time-of-flight mass spectrometry (UPLC-ESI-QTOF-MS/MS) has been applied to characterize the chemical composition of BCE.

## Materials and methods

### Preparation of *B. ceiba* flower aqueous extracts

*Bombax ceiba* flowers were obtained from Baise, Guangxi, China, in September 2020, and identified by Bin Yang. A voucher specimen (20200902) was deposited in Guangxi Medical University. The pure water was used to extract the shade dried *B. ceiba* flowers (1 kg). The filtrates were combined and then concentrated using a rotary evaporator. The excess water was removed by lyophilization with a freeze-dryer to obtain crude dark-brown sticky BCE for subsequent experiments. The yield of BCE from the extract was 10%.

### The analysis of BCE by UPLC-ESI-QTOF-MS/MS

BCE were analysed in negative and positive ionization mode by UPLC-ESI-QTOF-MS. Approximately, 0.5 g of the BCE were accurately weighed and ultrasonically extracted with 50 mL methanol/water (50%, v/v) for 30 min. Then, the extract was filtered through a 0.22 μm membrane prior to analysis. Identification of compounds in BCE was conducted on an Acquity UPLCH-Class system (Waters, Milford, MA) that was coupled to a Waters XevoG2-S QTof mass spectrometer (Waters, Milford, MA). Chromatographic separation was performed on a Waters ACQUITY UPLC column (BEH C18: 2.1 × 50 mm, 1.7 μm) by gradient elution at a flow rate of 0.3 mL/min. The mobile phase consisted of water containing 0.1% formic acid (A) and acetonitrile (B) with the following gradient procedure: 0–5 min, 5–20% B; 5–20 min, 20–65% B; 20–20.5 min, 65–90% B; 20.5–22 min, 90–5% B; then hold at 5% B for 3 min. The injection volume was 10 μL.

Mass spectrometry with an electrospray ionization (ESI) source was conducted in both the positive and negative ion modes. The analysis was performed using full scan mode, and the mass range was set at *m/z* 100–1000. The MS settings were the following: capillary voltage, 2.5 kV; sampling cone, 40 V; source temperature, 120 °C and desolvation temperature, 300 °C. A 10 eV collision energy was used during the MS acquisition, while 30 eV was employed during the MS^E^ acquisition. The setting for cone gas was 50 L/h. The data were gathered using MassLynxV4.1 software (Waters, Milford, MA).

### Animals

Male Kunming mice (20 ± 2 g) were provided by the Experimental Animal Center of Guangxi Medical University (Guangxi, China). All mice were housed in normal cages with relative humidity of 60 ± 10%, room air changes 12–18 times/h, temperature of 25 ± 2 °C and a 12 h light/dark cycle. All mice were allowed to have free access to standard chow pellets and water *ad libitum*. The animal experimental procedure was approved by the Institutional Animal Ethical Committee of Guangxi Medical University. The certificate number of animal care is SYXK GUI 2020-0004.

### Experimental design

Sixty mice were randomly divided into the following study groups (*n* = 10 each): a normal group (control): normal saline solution (0.25 mL)+normal saline solution (0.25 mL); a constipation model group (model): loperamide (lot: JEJ0944, Xian Janssen Pharmaceutical Ltd., Xi’an, China) (10 mg/kg; 0.25 mL)+normal saline solution (0.25 mL); a phenolphthalein: loperamide (10 mg/kg; 0.25 mL)+phenolphthalein (lot: 200502, Shandong Renhetang Pharmaceutical Co., Ltd., Shandong, China) (10 mg/kg; 0.25 mL); a low-dose BCE group (L-BCE): loperamide (10 mg/kg; 0.25 mL)+BCE (40 mg/kg; 0.25 mL); a medium-dose BCE group (M-BCE): loperamide (10 mg/kg; 0.25 mL)+BCE (80 mg/kg; 0.25 mL); a high-dose BCE group (H-BCE; 0.25 mL): loperamide (10 mg/kg; 0.25 mL)+BCE (160 mg/kg; 0.25 mL); the specific arrangements of the animal experiments are shown in Table 1.

**Table 1.** Specific arrangements of animal experiments.

Groups	Treatment (8 d)	
	Build model	Treatment (1 h later)
Control	Normal saline (0.25 mL)	Normal saline (0.25 mL)
Model	Loperamide (10 mg/kg; 0.25 mL)	Normal saline (0.25 mL)
Phenolphthalein	Loperamide (10 mg/kg; 0.25 mL)	Phenolphthalein (10 mg/kg; 0.25 mL)
L-BCE	Loperamide (10 mg/kg; 0.25 mL)	BCE (40 mg/kg; 0.25 mL)
M-BCE	Loperamide (10 mg/kg; 0.25 mL)	BCE (80 mg/kg; 0.25 mL)
H-BCE	Loperamide (10 mg/kg; 0.25 mL)	BCE (160 mg/kg; 0.25 mL)

The experiment was designed as described previously (Wang et al. 2017). All mice were fasted overnight (approximately 12 h) before the first experiment (with free access to water). Constipation was induced in animals via gavage of loperamide (10 mg per kg body weight loperamide hydrochloride in a volume of 0.25 mL) once per day for eight continuous days; 1 h later, the animals were administered with a corresponding concentration of BCE and phenolphthalein in the same manner. The animals in the normal group only received normal saline solution (a volume of 0.25 mL) by the same method.

### Faecal water content and number of faeces

The faecal water content and number of faeces were measured on day 7. At the end of the intragastric administration period, mice were moved into a clean separate cage. At 3 h, the faeces samples were collected in individual tubes on ice, counted and weighed; and then the samples thoroughly dried in an oven to obtain the dry weight. Faecal water content was calculated as:

$$\text{Faecal water content }(\%)=\frac{\text{Wet weight}-\text{dry weight}}{\text{Wet weight}}\times 100\%$$

### Measurement of first black faeces

At day 8, after fasting overnight 12 h with water provided, the mice in the normal group were received normal saline solution (0.25 mL) and those in other groups received an intragastric administration containing loperamide (10 mg/kg; 0.25 mL); 1 h later, all mice received an activated carbon meal by the same method. The mice were placed individually in a clean cage and allowed water and food *ad libitum*. The length of time from activated carbon meal to the appearance of first black faeces was recorded.

### Measurement of gastrointestinal transit rate and collection of blood and tissue sample

At day 9, the mice fasted 12 h but with free access to water and then fed 0.25 mL of activated carbon meal. At 1 h, serum samples were stored in −80 °C before further assays. The animal’s abdomen was opened. The total length of the small intestine (from the pyloric sphincter to the cecum) and the activated carbon transport distance were measured to characterize the gastrointestinal transit rate of the mice. Proximal colon tissues were collected and fixed in 10% formalin for subsequent histology evaluation and immunohistochemical (IHC) analysis. The intestinal transit rate was calculated based on the following equation:

$$\text{Intestinal transit rate }(\%)=\frac{\text{activated carbon transport distance }}{\text{total length of the small intestine}}\times 100\%$$

### Histology evaluation

Colon tissues were fixed in 10% formalin and embedded in paraffin. Then, they were cut 5 µm thick sections, deparaffinized in xylene and rehydrated in graded concentrations of ethanol. The sections were placed on glass slides. Then, they stained with haematoxylin–eosin (HE) and Alcian Blue/periodic acid Schiff (AB/PAS) according to the standard procedure. Pathological changes in the colon were examined under a light microscope (×100), and images were recorded.

### Assessment of MTL, gas, SP and SS levels in serum

The concentrations of MTL (lot: 2021-01, Jiangsu Jingmei Biological Technology Co., Ltd., Nanjing, China), Gas (lot: SB9JYJGPMA, Elabscience Biotechnology Co., Ltd., Wuhan, China), SP (lot: 2021-01, Jiangsu Jingmei Biological Technology Co., Ltd., Nanjing, China) and SS (lot: 2021-01, Jiangsu Jingmei Biological Technology Co., Ltd., Nanjing, China) in serum were estimated by enzyme-linked immunosorbent assay (ELISA) using commercially available kits.

### Immunohistochemical analysis

The pre-treatment of colon tissues for IHC examination of proteins expression (AQP_3_ and C-kit) was essentially the same as for histology evaluation. H_2_O_2_ (0.1%) was used to remove the endogenous peroxidase activity in the colon sections. Then, we incubated them with primary antibodies (1:200) (AQP_3_, lot: ab125219, Abcam, Cambridge, UK; C-kit, lot: ab256345, Abcam, Cambridge, UK) overnight at 4 °C and incubated them with a secondary antibody for 1 h. Finally, we counterstained the colon sections with haematoxylin. The sections were examined under a light microscope (×400). We calculated the expression area of AQP_3_ and C-kit by Image-Pro Plus 6.0 (Version X, Media Cybernetics, Silver Springs, MD).

### Statistical analysis

All statistical analyses were performed with GraphPad Prism 5 (GraphPad Software, La Jolla, CA). The data are presented as the mean ± standard deviation (SD) for each group. One-way analysis of variance (ANOVA) with Duncan’s multiple range tests was used to analyse the differences between the mean values of the groups. A *p* < 0.05 was considered to indicate statistical significance. The analyses were performed through SPSS 16.0 software (SPSS, Chicago, IL).

## Results

### Identification of the chemical composition in BCE

**Figure 1.**
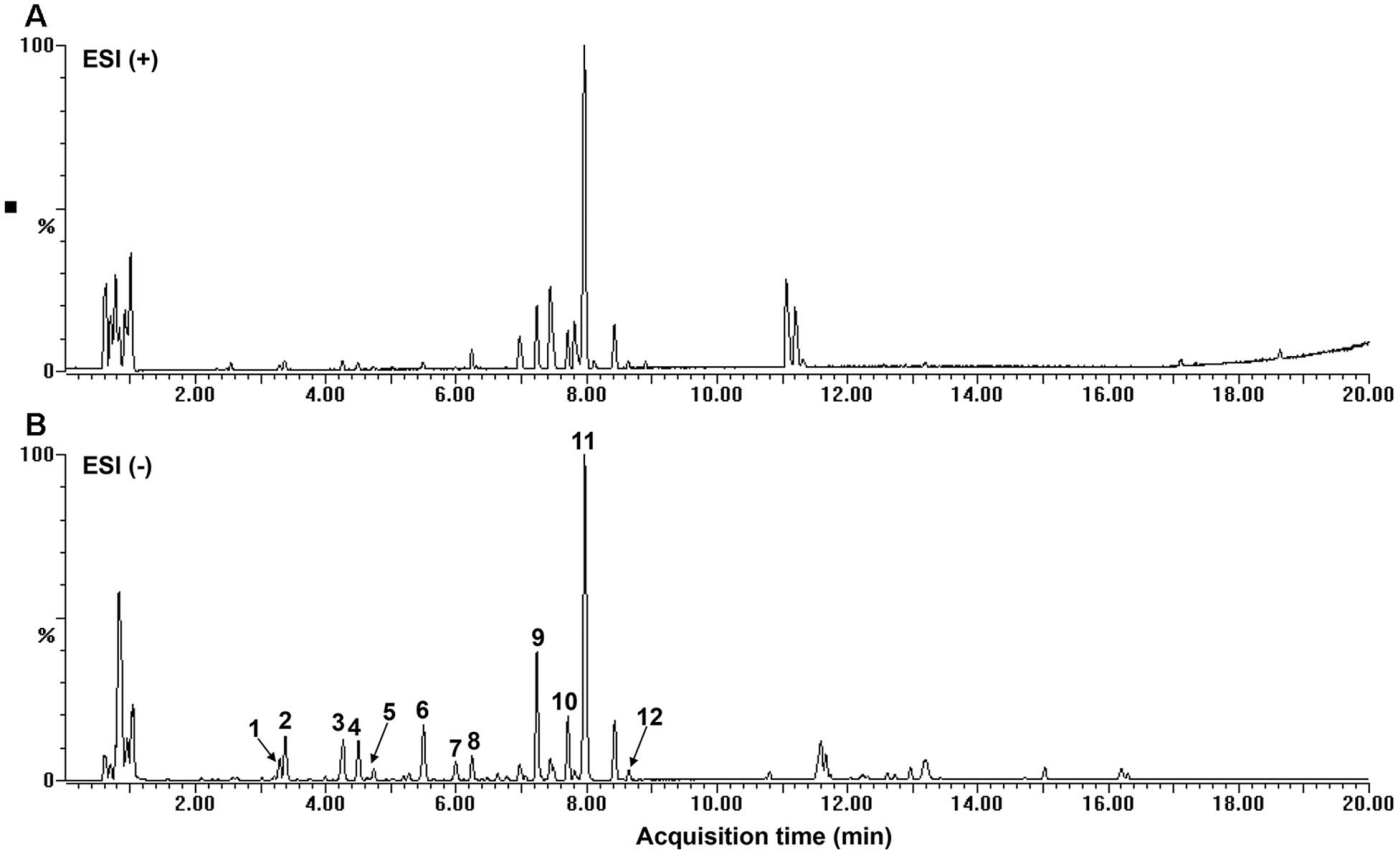
Base peak ion chromatogram (BPI). (A) Positive mode BPI. (B) Negative mode BPI of BCE by UPLC-ESI-QTOF-MS/MS.

In this study, the base peak chromatogram obtained is illustrated in Figure 1. The samples in negative ion mode showed stronger peak signals and rich mass information, so the peaks in negative ion mode we analysed to identification of the chemical composition in BCE. Based on the retention times, molecular formula, the MS/MS data, reference standards and literature data, 12 compounds were identified which include: protocatechuic acid, 1-caffeoylquinic acid, 5-coumaroylquinic acid, neochlorogenic acid, chlorogenic acid, 4-coumaroylquinic acid, 3-coumaroylquinic acid, clovamide, rutin, isoquercetin, quercetin 3-glucuronide and kaempferol-3-glucuronide (Table 2). Among them, three compounds, including protocatechuic acid, chlorogenic acid and rutin were unambiguously identified by comparing with the retention time and MS data of reference standards. In addition to the above compounds, some peaks of fatty acids were found in the chromatogram of BCE after 10 min.

**Table 2.** UPLC-QTOF-MS/MS data for the identification of components from BCE.

No.	*T*_R_ (min)	Formula	Ion	Experimental, *m/z*	Calculated, *m/z*	Fragment	Identification
**1**	3.276	C_7_H_6_O_4_	[M–H]–	153.0837	153.0188	109.0887	Protocatechuic acid
**2**	3.38	C_16_H_18_O_9_	[M–H]–	353.1612	353.0873	191.1243; 179.1142; 135.1089	1-Caffeoylquinic acid
**3**	4.261	C_16_H_18_O_8_	[M–H]–	337.1653	337.0923	191.1243; 163.1058	5-Coumaroylquinic acid
**4**	4.497	C_16_H_18_O_9_	[M–H]–	353.1612	353.0873	191.1243; 173.1142; 135.1089	Neochlorogenic acid
**5**	4.723	C_16_H_18_O_9_	[M–H]–	353.1574	353.0873	191.1243; 177.0882; 133.00938	Chlorogenic acid
**6**	5.496	C_16_H_18_O_8_	[M–H]–	337.1653	337.0923	173.1142; 163.1089	4-Coumaroylquinic acid
**7**	5.989	C_16_H_18_O_8_	[M–H]–	337.1653	337.0923	191.1243; 173.1142; 163.1089	3-Coumaroylquinic acid
**8**	6.238	C_18_H_17_NO_7_	[M–H]–	358.1687	358.0927	178.1194; 161.0910; 135.1089	Clovamide
**9**	7.267	C_27_H_30_O_16_	[M–H]–	609.2032	609.1456	300.1008	Rutin
**10**	7.716	C_21_H_20_O_12_	[M–H]–	463.1568	463.0877	300.1008; 271.0973; 255.1037	Isoquercetin
**11**	7.981	C_21_H_18_O_13_	[M–H]–	477.139	477.0669	301.1089; 271.0973; 151.0691	Quercetin 3-glucuronide
**12**	8.644	C_21_H_18_O_12_	[M–H]–	461.1412	461.072	285.114	Kaempferol-3-Glucuronide

### Effect of BCE on faeces number and moisture content in loperamide-induced constipation mice

Compared with the control group, the faeces number and moisture content within 3 h in the model group were significantly decreased (*p* < 0.01) (Table 3). When treated by BCE, the faeces number in the L-BCE group and M-BCE group were increased (*p* < 0.05). Moreover, after treatment with L-BCE, M-BCE, H-BCE and phenolphthalein, the water content levels were increased compared with the model group (*p* < 0.05).

**Table 3.** The faecal water content, number of faeces, defecating time of first black faeces and intestinal transit rate in loperamide-induced constipation mice after treatment.

Groups	Faecal water content (%)	Number of faeces (granule)	Defecating time of first black faeces (min)	Intestinal transit rate (%)
Control	70.21 ± 5.04	13.10 ± 1.79	101.10 ± 21.86	75.28 ± 12.12
Model	56.36 ± 8.50^##^	9.10 ± 2.96^##^	436.70 ± 60.30^##^	47.74 ± 11.62^##^
Phenolphthalein	64.28 ± 7.28^#,^**	10.70 ± 2.63	383.90 ± 57.17^##^	52.97 ± 10.18^##^
L-BCE	62.81 ± 7.19^#,^*	12.50 ± 2.46*	348.40 ± 82.87^##^	54.67 ± 10.59^##^
M-BCE	63.90 ± 5.14^#,^*	12.30 ± 4.06*	343.90 ± 63.65^##,^*	50.18 ± 13.49^##^
H-BCE	66.90 ± 5.52**	10.30 ± 4.03^#^	331.70 ± 57.79^##,^*	63.97 ± 8.05^#,^**

^#^*p* < 0.05 or ^##^*p* < 0.01 compared to the control group, *p < 0.05 or **p < 0.01 compared to the model group, *n* = 10 per group.

### Effect of BCE on first black excretion time in loperamide-induced constipation mice

The time to the first black excretion time was significantly prolonged in the model group compared with the control group (*p* < 0.01). After treatment with M-BCE and H-BCE, the first black excretion time was decreased compared with the model group (*p* < 0.05) (Table 3).

### Effect of BCE on intestinal transit rate in loperamide-induced constipation mice

**Figure 2.**
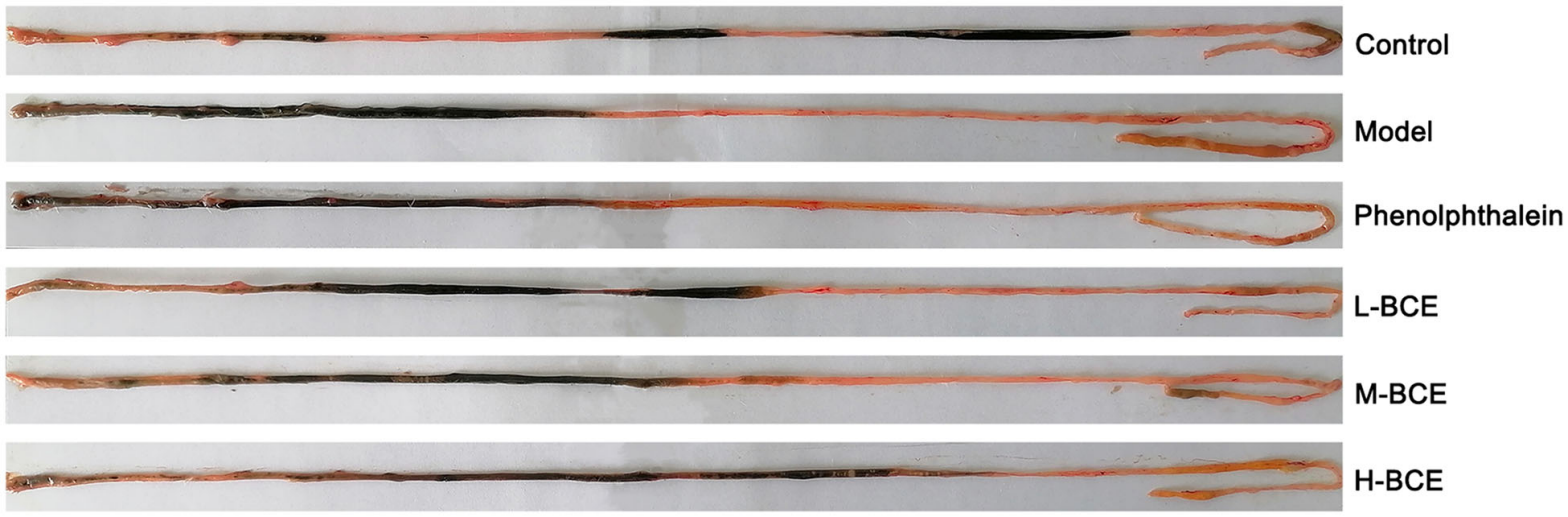
BCE increased the carbon propulsion rate in loperamide-induced constipation mice. Mice were given carbon and then the small intestine was taken out 30 min later.

As shown in Table 3 and Figure 2, the intestinal transit ratio significantly decreased in the model group compared with the control group (*p* < 0.01). After treatment with BCE, the intestinal transit ratio increased in the H-BCE group compared with the model group (*p* < 0.05).

### Histopathological alterations of colon

**Figure 3.**
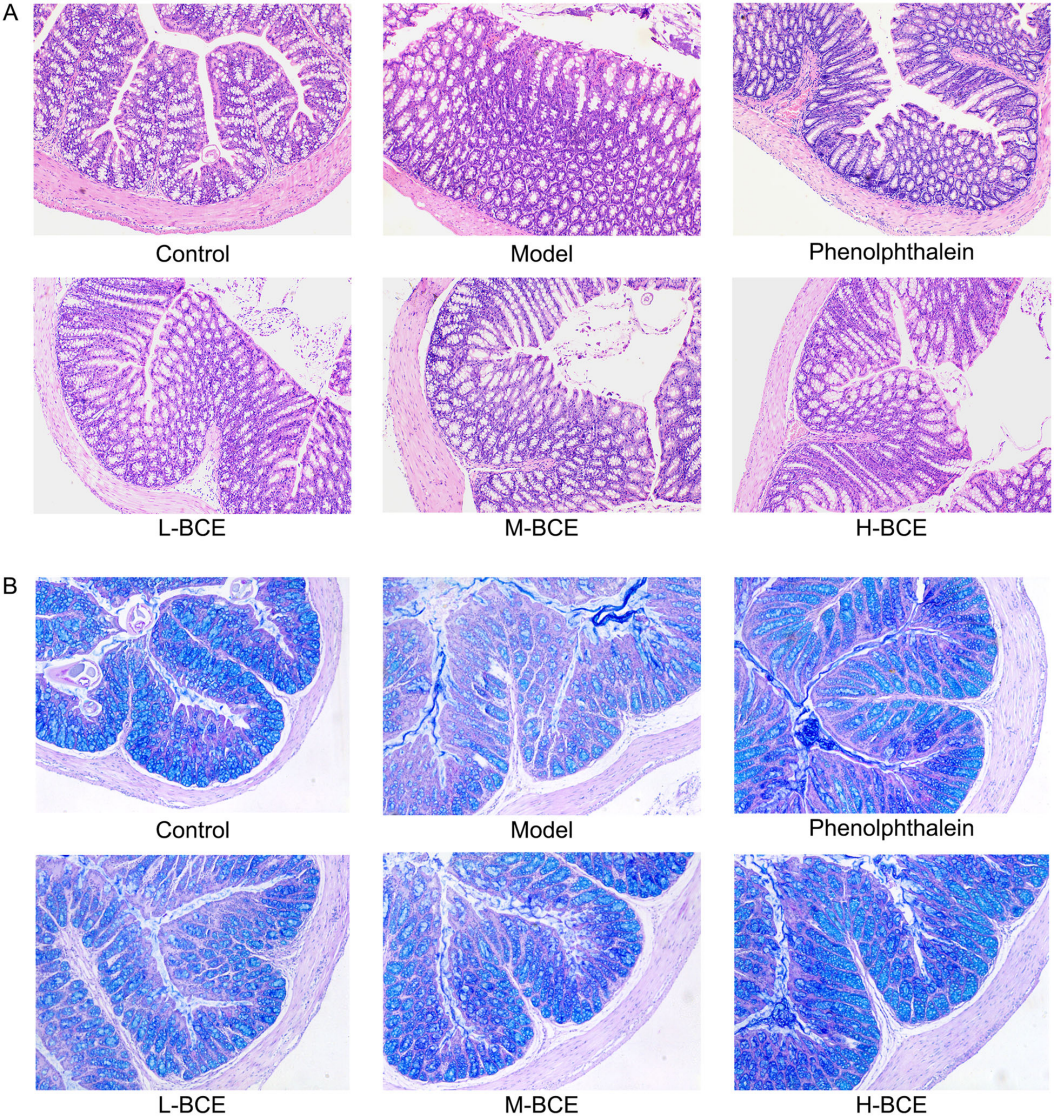
BCE attenuated histopathological changes in colon of loperamide-induced constipation mice. (A) Representative H&E staining images of mice, ×100. *n* = 4 per group. (B) Representative AP/PAS (blue indicates mucin) staining images of mice, ×100. *n* = 4 per group.

Figure 3(A) shows the histological alterations of the mouse colonic tissue with loperamide-induced constipation by H&E and AB/PAS staining. The animals treated with loperamide alone exhibited a loss of epithelium and goblet cells depletion of the colon compared with the control group (Figure 3(A,B)). Following treatment, mucosal damage was reduced when compared with the model group (Figure 3(A)). The colons of mice in the phenolphthalein and BCE (L-BCE, M-BCE and H-BCE) group restored the secretory activity of goblet cells, which were comparable to those of the model group (Figure 3(B)).

### Parameters of serum

To evaluate the effects of BCE on serum biochemical components in the loperamide-induced constipation mice, alterations of several components related to gastrointestinal motility-related biomarkers in serum were assessed by ELISA. As shown in Table 4, the levels of MTL, Gas and SP were remarkably decreased in the model group compared with the control group (*p* < 0.01), while SS was increased although the effect was not noteworthy. With the intervention of BCE or phenolphthalein, MTL, Gas and SP were increased but SS was decreased compared with the model group (*p* < 0.05 or not noteworthy) (Table 4). Therefore, these results show that BCE treatment may regulate the biomarkers related to gastrointestinal motility to relieve loperamide-induced constipation in Kunming mice.

**Table 4.** The concentrations of MTL, Gas, SP and SS in loperamide-induced constipation mice after treatment.

Groups	MTL (pg/mL)	Gas (pg/mL)	SP (pg/mL)	SS (pg/mL)
Control	511.99 ± 139.71	126.89 ± 40.73	176.65 ± 39.61	24.70 ± 2.37
Model	312.82 ± 94.15^##^	65.42 ± 53.15^##^	117.41 ± 20.75^##^	26.19 ± 1.92
Phenolphthalein	430.47 ± 96.76**	85.45 ± 46.28^#^	137.64 ± 32.40^##^	23.40 ± 1.23*
L-BCE	421.66 ± 57.32^#,^*	93.65 ± 34.81^#^	135.73 ± 22.77^##^	22.60 ± 2.81*
M-BCE	450.90 ± 75.38**	143.66 ± 49.56**	144.94 ± 30.03^#,^*	21.09 ± 4.05*
H-BCE	410.40 ± 89.41^#,^*	108.41 ± 25.58*	154.34 ± 32.65**	21.57 ± 2.50**

^#^*p* < 0.05 or ^##^*p* < 0.01 compared to the control group, *p < 0.05 or **p < 0.01 compared to the model group, *n* = 10 per group.

### Effect of BCE on the expression of AQP_3_ and C-kit in loperamide-induced constipation mice

**Figure 4.**
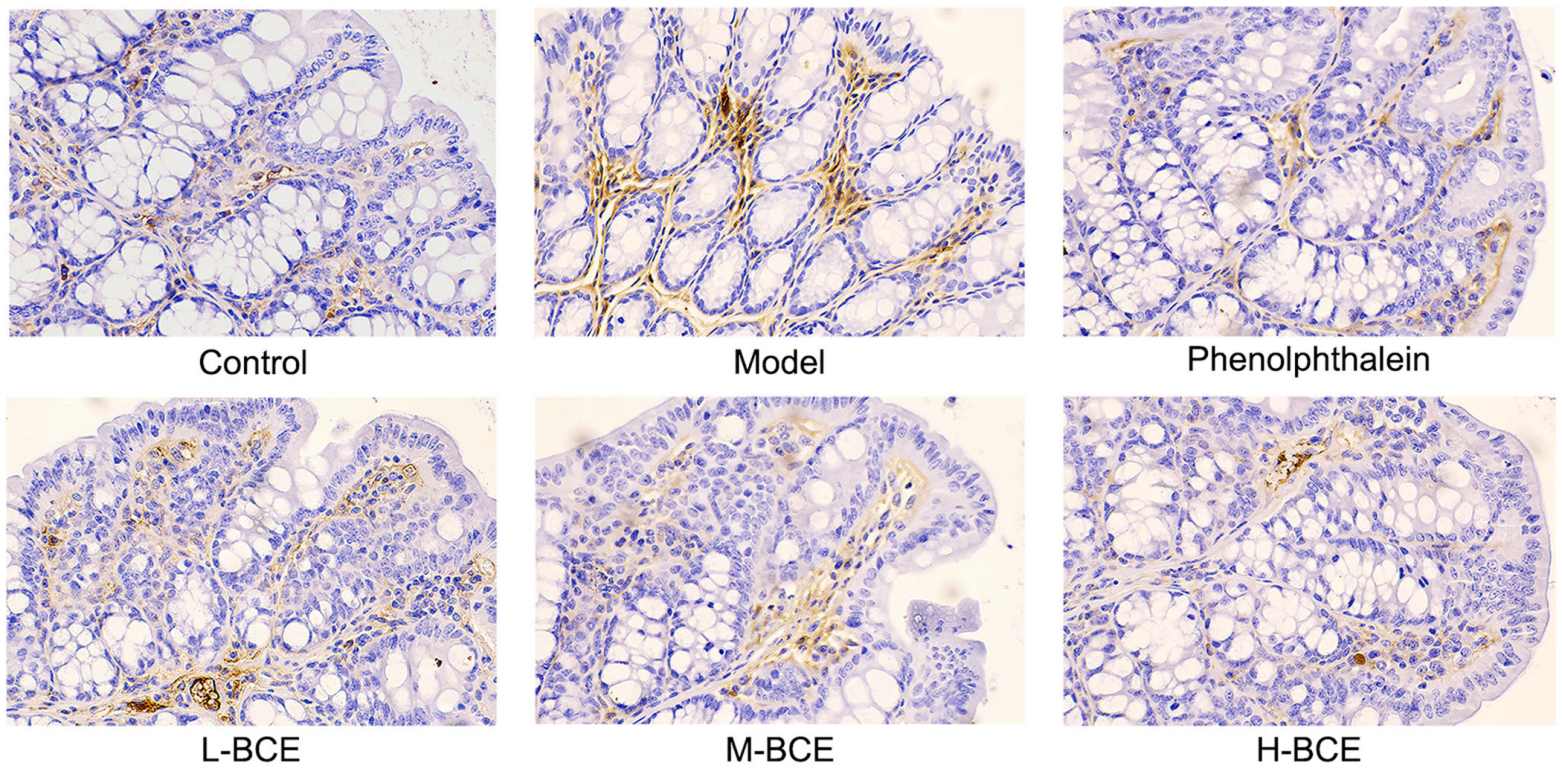
BCE reduced the expression of AQP_3_ in colon. Protein expression of AQP_3_ in the colon was identified by immunohistochemical analysis, ×400. *n* = 10 per group.

**Figure 5.**
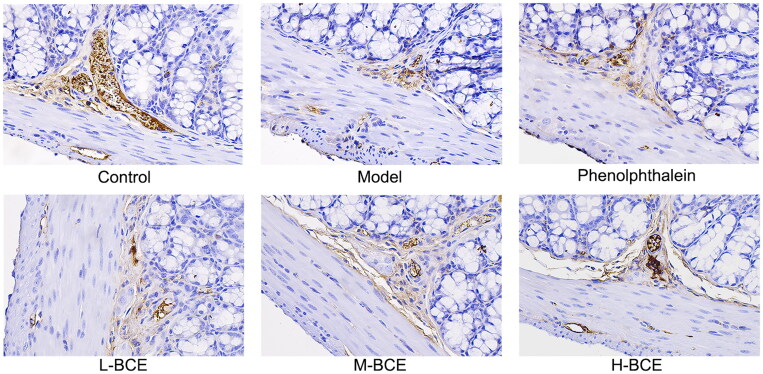
BCE improved the expression of C-kit in colon. Protein expression of C-kit in the colon was identified by immunohistochemical analysis, ×400. *n* = 10 per group.

AQP_3_ plays an important role in regulating water transport effect in the colon. Moreover, ICC is a pacemaker of the intestine that plays an important role in controlling intestinal movement. Thus, we investigated the effect of BCE on the expression of AQP_3_ and C-kit in the colons of Kunming mice with loperamide-induced constipation. As shown in Table 5 and Figure 4, the levels of AQP_3_ were increased in the model group compared with the control group (*p* < 0.05), whereas the levels of AQP_3_ were decreased in the BCE and phenolphthalein group compared with the model group (*p* < 0.05 or *p* < 0.01). C-kit expression was decreased in the mice of model group compared with the control group (*p* < 0.05). Interestingly, the C-kit expression was remarkably increased in H-BCE group (*p* < 0.01) (Table 5 and Figure 5).

**Table 5.** The protein expression of AQP_3_ and C-kit in the colon of loperamide-induced constipation mice.

Groups	Integral optical density (IOD)	
	AQP_3_	c-Kit
Control	40.53 ± 7.56	61.07 ± 5.99
Model	67.38 ± 6.69^##^	48.31 ± 5.35^##^
Phenolphthalein	53.10 ± 4.79^##,^**	50.95 ± 5.25^##^
L-BCE	61.42 ± 4.68^##^	49.96 ± 4.85^##^
M-BCE	56.30 ± 6.69^##,^*	52.03 ± 5.77^##^
H-BCE	44.74 ± 2.76**	54.67 ± 6.75^#,^*

^#^*p* < 0.05 or ^##^*p* < 0.01 compared to the control group, *p < 0.05 or **p < 0.01 compared to the model group, *n* = 10 per group.

## Discussion

Constipation is a common digestive tract disease (Liu et al. 2020) mainly caused by intestinal disorders, which is associated with infrequent bowel movements (Yin et al. 2018), altered bowel habits, difficulty during defecation and disappearance of defecation intention that causes discomfort and seriously affect the quality of life of patients (Wang et al. 2017). Some studies suggest that multiple plant extracts are attracting attention nowadays because of their laxative and there is hardly any side effect on constipation (Gilani et al. 2000; Han 2015; Lu et al. 2021). Moreover, according to the traditional Chinese medicine theory, the *B. ceiba* flower is cool-natured (Zhang et al. 2015), and is regarded as having laxative property (Shahat et al. 2003). BCE is an aqueous extract from *B. ceiba* flower, and our study suggests that BCE has a positive effect on relieving the symptoms of loperamide-induced constipation mice.

Loperamide, as an opioid receptor agonist (Hayeeawaema et al. 2020), is well known to decrease smooth muscle contraction and relaxation during intestinal peristalsis and delay gastrointestinal transit time (Wintola et al. 2010; Nelson and Camilleri 2015). Furthermore, loperamide has been used to induce spastic constipation (Wintola et al. 2010) in a variety of experiments to reveal the cause of constipation and determine novel compounds with therapeutic effects (Kakino et al. 2010; Lee et al. 2012; Yin et al. 2018). In the present study, the loperamide (model) group showed evident constipation symptoms, including significant decreases in faecal water content, faecal pellets number and small intestine transit rate and significant increases the time to the first blank faeces defecation in comparison to the control group. Moreover, there was no death in animals during the process of the study. This proves that loperamide could successfully induce the model of constipation which was relatively safe.

A common factor of constipation is that stool take longer to pass through the gastrointestinal tract, which cause stool to lose water and become hard-dry stool (Li et al. 2014). The transport function of the whole intestine could be reflected by the detection of the first black-stool defecation time (Lu et al. 2021), which is the sum time of the large intestine transit and the small intestine transit (Wang et al. 2017). A shorter time to the first black-stool defecation indicates a better effect of treatment and the prognosis; otherwise, the effect will be worse (Wang et al. 2017). Moreover, the intestinal transit rates were used to measure the small intestine transit time (Lu et al. 2021). A smaller value to the intestinal transit rates shows a better effect of treatment and the prognosis. The present study suggests that amount of defecation, moisture content of faeces, the intestinal transit rates, and the first black-stool defecation time have significantly changed in model of loperamide-induced constipation mice, while BCE could improve those parameters. In addition, phenolphthalein could only improve moisture content of faeces.

As it is known that mucins play an important role in the protection of the gastrointestinal epithelium, and quantitative change in mucin secretion might modify gastrointestinal defensive barrier (Wu et al. 2019). Moreover, most mucin is secreted by goblet cells in the gastrointestinal tract, which is a diverse family of densely glycosylated proteins with its characteristic ability to form gels, protect gastrointestinal tract and facilitate intestinal movement (Furness et al. 2013; Van Spaendonk et al. 2017). The results of our study indicated that BCE could restore the secretory activity of goblet cells. However, the precise mechanisms remain to be further researched.

The gastrointestinal motility-related biomarkers secreted by the intestinal nerve network act as neuromodulators and neurotransmitters to promote intestinal peristalsis and transportation, including excitatory factors (MTL, SP, Gas, etc.) (Yin et al. 2018) and inhibitory factor (SS, NO, etc.) (Gan et al. 2020). Abnormal secretion of enteric motility-related biomarkers may be the main pathogenesis of constipation (Liu et al. 2020). MTL is the intestinal hormone which can increase the migrating motor complex of gastrointestinal motility (Yin et al. 2018), but it has lower serum MTL levels in constipation children (Sun et al. 2020). As an excitatory neurotransmitter of intestinal nerve network, SP strongly promotes smooth muscle contraction, promotes gastrointestinal peristalsis and stimulates intestinal water and electrolyte secretion (Li et al. 2020). Gas, mainly secreted by G cells in the pyloric antrum of the stomach (Iijima et al. 2014), promotes gastrointestinal motility and stimulates the secretion of gastric acid by the parietal cells of the stomach (Gan et al. 2020). However, the secretion of Gas is inhibited by SS (Jiang et al. 2020). SS could suppress the movement of gastrointestinal smooth muscle and inhibit the secretion of gastrointestinal hormones (Han 2013). According to current ELISA results, with the intervention of phenolphthalein, MTL was increased but SS was decreased compared with the model group. In addition, compared with the model group, BCE significantly increased levels of serum MTL, SP, Gas, while inhibited serum SS. Nevertheless, the concrete relationship and mechanism between them still need further research.

Aquaporins are mainly expressed in the intestinal canal (Sun et al. 2020), which mediate the passive transport of free water across biofilm, thereby maintaining the homeostasis of the intracellular and extracellular environment (Matsuzaki et al. 2004). Moreover, the abnormal expression of aquaporins in the gastrointestinal tract is related to the occurrence of some diseases, such as constipation, gastritis, diarrhoea and gastric cancer (Liu et al. 2020). AQP_3_ is an important aquaporin located in the colon and is permeable to water (Li and Wang 2017). Previously studies have revealed the relationship between AQP_3_ and constipation, particularly morphine-induced constipation (Kon et al. 2015), promotes AQP_3_ expression level in the colon and subsequently increases water absorption from the luminal side to vasculature, which dries and hardens stool (Ikarashi et al. 2016). In the present study, the expression level of AQP_3_ was detected and the results showed that phenolphthalein and BCE relieve the symptoms of loperamide-induced constipation by decreasing the level of AQP_3_ in the colon of mice. However, the precise mechanisms remain to be further elucidated.

Interstitial cells of Cajal, as the gastrointestinal pacemaker cells (Yin et al. 2018), may play an important role in regulating neurotransmitters (Lees-Green et al. 2011), producing the smooth muscle electrical slow wave and conducting slow-wave potential, thereby coordinating intestinal motility (Xu et al. 2013). Moreover, the slow wave appears to be generated by submucosal ICC in the colon, which determines smooth muscle contractile activity (Li et al. 2015). The significant decrease of ICC may produce abnormal slow wave (Li et al. 2015), which can inhibit smooth muscle contractile activity and disrupt normal colonic motility, resulting in constipation (Su et al. 2019). As tyrosine kinase receptors of ICC, C-kit is critical in the maintenance of the ICC network and loss of C-kit expression may suggest the disruption of the ICC network in patients with constipation (Li et al. 2015). C-kit can be reliably identified by IHC techniques (Yin et al. 2018), which indirectly reflects the quantity and density of ICC (Liu et al. 2020). The results of our study indicated that BCE could increase the expression of c-kit protein, which protect the expression of ICC. But its mechanism might need further research in the future.

Nowadays, researchers have isolated and identified a variety of monomeric compounds from *B. ceiba* flower, including flavonoids, phenylpropanoid and phenolic acid compounds. Such as alanopine, angoletin (Komati et al. 2022), quercetin 3-glucuronide, rutin, neochlorogenic acid, 4-coumaroylquinic acid, caffeic acid, ferulic acid, syringic acid, protocatechuic acid, etc. (El-Hagrassi et al. 2011; Joshi et al. 2013; Zhang et al. 2015). In this article, 12 components in BCE were detected by UPLC-ESI-QTOF-MS. Among the 12 components, protocatechuic acid, chlorogenic acid, rutin and isoquercetin showed promising pharmacological activities in constipation (Rtibi et al. 2019), smooth muscle-relaxing (Tóth et al. 2015) and anti-inflammatory (Ezenyi et al. 2018).

Notably, except for constipation, *B. ceiba* is also traditionally used for the treatment of diarrhoea (Zhang et al. 2015), but scientific basis and study for this effect have not been provided (Yu et al. 2011; Arafa et al. 2019). Similar dual efficacy for diarrhoea and constipation has been reported for some popular herbs, such as Ginger and *Psyllium* husk (Mehmood et al. 2011), which is probably meant by the nature to offset the side-effects, such as diarrhoea and constipation usually observed with antidiarrheal chemical agents and laxative respectively when used alone at high doses (Palla and Gilani 2015). It may fit in the philosophy that natural products should be valued as a whole (Ahmed and Gilani 2014), as they have multiple targets to address disease along with having side-effect neutralizing potential (Gilani and Rahman 2005). However, further research on the antidiarrheal efficacy of BCE is needed.

## Conclusions

BCE significantly increased the water content of faeces after constipation modelling, and BCE at a high‐dose exhibited the best effect for constipation relief. BCE could increase the number of faeces and promote small intestinal transit. The same tendency was observed for the time to the first black stool defecation. The potential mechanism may be associated with restoring the secretory activity of goblet cells, regulating the content of intestinal hormones and reducing the expression of AQP_3_ in the colon. In addition, BCE might improve the slow-wave potential of colon and regulate smooth muscle contractile activity by up-regulating c-kit in ICC. The extracts contained valuable flavonoids, and phenylpropanoids, they were likely the active compounds contributing to the therapeutic effect of BCE. These results suggested that BCE may be effective as a candidate in patients suffering from constipation.
